# Recent Advances in the Modeling of Alzheimer’s Disease

**DOI:** 10.3389/fnins.2022.807473

**Published:** 2022-03-31

**Authors:** Hiroki Sasaguri, Shoko Hashimoto, Naoto Watamura, Kaori Sato, Risa Takamura, Kenichi Nagata, Satoshi Tsubuki, Toshio Ohshima, Atsushi Yoshiki, Kenya Sato, Wakako Kumita, Erika Sasaki, Shinobu Kitazume, Per Nilsson, Bengt Winblad, Takashi Saito, Nobuhisa Iwata, Takaomi C. Saido

**Affiliations:** ^1^Laboratory for Proteolytic Neuroscience, RIKEN Center for Brain Science, Wako, Japan; ^2^Laboratory for Molecular Brain Science, Department of Life Science and Medical Bioscience, Waseda University, Shinjuku City, Japan; ^3^Department of Functional Anatomy and Neuroscience, Nagoya University Graduate School of Medicine, Nagoya, Japan; ^4^Experimental Animal Division, RIKEN BioResource Research Center, Tsukuba, Japan; ^5^Department of Marmoset Biology and Medicine, Central Institute for Experimental Animals, Kawasaki, Japan; ^6^Laboratory for Marmoset Neural Architecture, RIKEN Center for Brain Science, Wako, Japan; ^7^Department of Clinical Laboratory Sciences, School of Health Sciences, Fukushima Medical University, Fukushima, Japan; ^8^Division of Neurogeriatrics, Department of Neurobiology, Care Sciences and Society, Bioclinicum, Karolinska Institutet, Stockholm, Sweden; ^9^Department of Neurocognitive Science, Institute of Brain Science, Nagoya City University Graduate School of Medical Sciences, Nagoya, Japan; ^10^Department of Neuroscience and Pathobiology, Research Institute of Environmental Medicine, Nagoya University, Nagoya, Japan; ^11^Department of Genome-Based Drug Discovery and Leading Medical Research Core Unit, Graduate School of Biomedical Sciences, Nagasaki University, Nagasaki, Japan

**Keywords:** Alzheimer’s disease, amyloid – beta, amyloidosis, tau propagation, somatostatin, mouse model, non-human primate (NHP)

## Abstract

Since 1995, more than 100 transgenic (Tg) mouse models of Alzheimer’s disease (AD) have been generated in which mutant amyloid precursor protein (APP) or APP/presenilin 1 (PS1) cDNA is overexpressed (***1st generation models***). Although many of these models successfully recapitulate major pathological hallmarks of the disease such as amyloid β peptide (Aβ) deposition and neuroinflammation, they have suffered from artificial phenotypes in the form of overproduced or mislocalized APP/PS1 and their functional fragments, as well as calpastatin deficiency-induced early lethality, calpain activation, neuronal cell death without tau pathology, endoplasmic reticulum stresses, and inflammasome involvement. Such artifacts bring two important uncertainties into play, these being (1) why the artifacts arise, and (2) how they affect the interpretation of experimental results. In addition, destruction of endogenous gene loci in some Tg lines by transgenes has been reported. To overcome these concerns, single *App* knock-in mouse models harboring the Swedish and Beyreuther/Iberian mutations with or without the Arctic mutation (*App^NL–G–F^* and *App^NL–F^* mice) were developed (***2nd generation models***). While these models are interesting given that they exhibit Aβ pathology, neuroinflammation, and cognitive impairment in an age-dependent manner, the model with the Artic mutation, which exhibits an extensive pathology as early as 6 months of age, is not suitable for investigating Aβ metabolism and clearance because the Aβ in this model is resistant to proteolytic degradation and is therefore prone to aggregation. Moreover, it cannot be used for preclinical immunotherapy studies owing to the discrete affinity it shows for anti-Aβ antibodies. The weakness of the latter model (without the Arctic mutation) is that the pathology may require up to 18 months before it becomes sufficiently apparent for experimental investigation. Nevertheless, this model was successfully applied to modulating Aβ pathology by genome editing, to revealing the differential roles of neprilysin and insulin-degrading enzyme in Aβ metabolism, and to identifying somatostatin receptor subtypes involved in Aβ degradation by neprilysin. In addition to discussing these issues, we also provide here a technical guide for the application of *App* knock-in mice to AD research. Subsequently, a new double knock-in line carrying the *App^NL–F^* and *Psen1*^*P*117*L/WT*^ mutations was generated, the pathogenic effect of which was found to be synergistic. A characteristic of this ***3rd generation model*** is that it exhibits more cored plaque pathology and neuroinflammation than the *App^NL–G–F^* line, and thus is more suitable for preclinical studies of disease-modifying medications targeting Aβ. Furthermore, a derivative *App^G–F^* line devoid of Swedish mutations which can be utilized for preclinical studies of β-secretase modifier(s) was recently created. In addition, we introduce a new model of cerebral amyloid angiopathy that may be useful for analyzing amyloid-related imaging abnormalities that can be caused by anti-Aβ immunotherapy. Use of the *App* knock-in mice also led to identification of the α-endosulfine-K_*ATP*_ channel pathway as components of the somatostatin-evoked physiological mechanisms that reduce Aβ deposition *via* the activation of neprilysin. Such advances have provided new insights for the prevention and treatment of preclinical AD. Because tau pathology plays an essential role in AD pathogenesis, knock-in mice with human tau wherein the entire murine *Mapt* gene has been humanized were generated. Using these mice, the carboxy-terminal PDZ ligand of neuronal nitric oxide synthase (CAPON) was discovered as a mediator linking tau pathology to neurodegeneration and showed that tau humanization promoted pathological tau propagation. Finally, we describe and discuss the current status of mutant human tau knock-in mice and a non-human primate model of AD that we have successfully created.

## 1st, 2nd, and 3rd Generation Mouse Models of Alzheimer’s Disease

**FIGURE 1:**
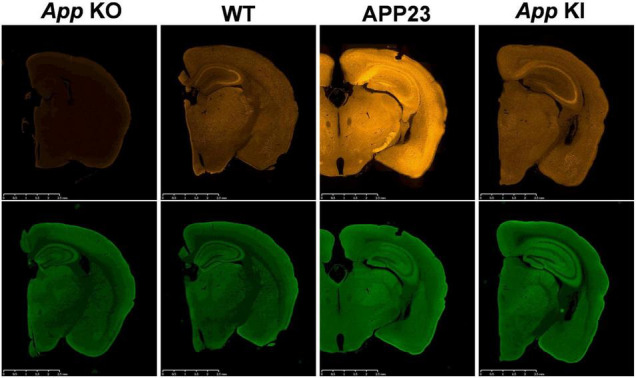
Mislocalization of APP in APP-overexpressing mice. *App* KO mice, WT mice, APP23 (APP-overexpressing mice) and *App* KI mice (*App^NL–F/NL–F^*) were subjected to immunohistochemistry using antibodies to APP, 22C11 **(upper panels)** and synaptophysin, a synaptic vesicle marker **(lower panels)** as indicated. *App* KO mice were used as negative controls for APP staining. While APP is selectively expressed in the axons of WT and KI mice, APP23 expresses unphysiologically high levels of APP not only in the axons but also in the soma and dendrites. The scale bar indicates 2 mm.

The deposition of amyloid β peptide (Aβ) in the brain is the major pathological hallmark of Alzheimer’s disease (AD), which is considered the most common type of dementia in the world (Karran and De Strooper, 2016; Selkoe and Hardy, 2016). To date, disease-associated mutations in the *presenilin 1* (*PSEN1*) and *presenilin 2* (*PSEN2*) genes number more than 300, while more than 50 mutations have been reported in the *amyloid precursor protein* (*APP*) gene (Alzforum^1^). In response to these findings, many transgenic mouse models overexpressing mutant APP or APP/PSEN1 cDNAs have been developed (**1st generation models**) (Sasaguri et al., 2017), however they often suffer from experimental limitations resulting from the mislocalization of APP (Figure 1) and by the overproduction of APP fragments such as the C-terminal fragment of APP generated by β-secretase (CTF-β) and APP intracellular domain (AICD). Neither of these fragments appears to accumulate in AD brains, meaning that artificial endosomal abnormalities (Kwart et al., 2019) and transcriptional malfunctions (Nalivaeva et al., 2014), respectively, may be induced. Other overexpression artifacts include calpain activation (Saito et al., 2016), calpastatin deficiency-induced early lethality (Higuchi et al., 2012), and endoplasmic reticulum stresses (Hashimoto et al., 2018). Furthermore, it was demonstrated that the random insertion of transgene(s) resulted in the destruction of unexpectedly large regions of endogenous gene loci in the host animal (Gamache et al., 2019). We suggest that all transgenic mouse models being used in research in which APP or APP/PSEN1 are overexpressed should undergo whole genome sequencing (WGS) so that destroyed loci that possibly affect their phenotypes can be identified (Sasaguri et al., 2017).

**FIGURE 2:**
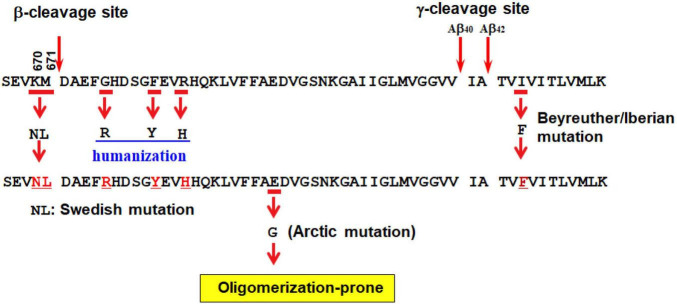
Second generation mouse models of Alzheimer’s disease. See text for detailed explanation.

To overcome these drawbacks, single *App* knock-in mice, i.e., *App^NL–G–F/NLG–F^* knock-in (*App^NL–G–F^*) and *App^NL–F/NL–F^* knock-in (*App^NL–F^*) lines, were generated that harbor the Swedish (KM670/671NL) (Citron et al., 1992; Mullan et al., 1992) and Beyreuther/Iberian (I716F) (Lichtenthaler et al., 1999) mutations with or without the Arctic (E693G) (Nilsberth et al., 2001) mutation (**2nd generation models**) (Figure 2) (Saito et al., 2014; Sasaguri et al., 2017). These mice, which exhibit typical Aβ pathology, neuroinflammation and memory impairment (Saito et al., 2014; Masuda et al., 2016), are being used in more than 500 research laboratories world-wide. At present, the *App^NL–G–F^* line is being used more frequently than the *App^NL–F^* line given that it develops Aβ pathology approximately three times faster (Saito et al., 2014) and can be used to analyze downstream events such as neuroinflammation (Shirotani et al., 2019; Chen et al., 2020; Sobue et al., 2021), pericyte signaling (Nortley et al., 2019), oxidative stress (Hashimoto et al., 2019; Hongo et al., 2020; Uruno et al., 2020), tau propagation (Saito et al., 2019), and spatial memory impairment (Masuda et al., 2016; Jun et al., 2020; Sutoko et al., 2021; Table 1). Human Arctic mutation carriers are indistinguishable from other familial and sporadic AD patients in pathological and neurological terms except for low retention of ^11^C-labeled Pittsburgh compound B (PiB) in PET study (Basun et al., 2008), indicating that the mutant mice are relevant models for studying AD in general.

**TABLE 1:** Successful application of the 2nd generation mouse models.

(1) Behavioral analysis using IntelliCage (Masuda et al., 2016; Sutoko et al., 2021).
(2) Three-dimensional visualization of amyloid pathology by transparency (Hama et al., 2015; Susaki et al., 2020).
(3) Impairment of gamma oscillations in medial entorhinal cortex (Nakazono et al., 2017).
(4) Additional genetic manipulation of the 2nd generation models through genome editing (Nagata et al., 2018; Watamura et al., 2021b).
(5) Generation of the double knock-in mouse models (Hashimoto et al., 2019; Saito et al., 2019; Sato et al., 2021).
(6) Assessment of vascular dysfunction in the 2nd generation models (Nortley et al., 2019; Tachida et al., 2020).
(7) Assessment of sleep dysfunction in the 2nd generation models (Maezono et al., 2020).
(8) Assessment of place cell dysfunction in the 2nd generation models (Jun et al., 2020; Takamura et al., 2021).
(9) Analyses of various aspects of neuroinflammation (Shirotani et al., 2019; Chiasseu et al., 2020; Salobrar-García et al., 2020; Barrett et al., 2021; Sobue et al., 2021).
(10) Application of spatial transcriptomics (Chen et al., 2020).

**FIGURE 3:**
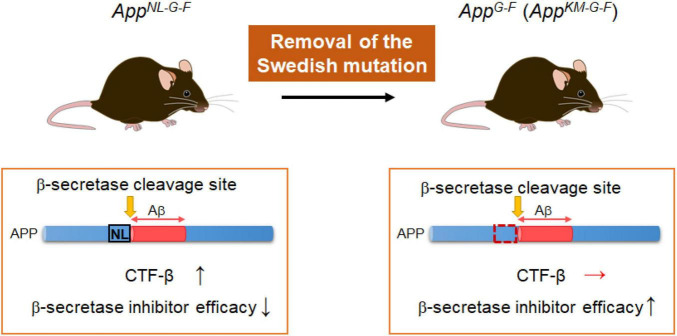
*App^G–F^* mice suitable for studies of BACE1 inhibitors. The *App^G–F^* line is devoid of the Swedish mutation that influences the β-secretase activity and elevates the quantity of CTFβ. (The *App^G–F^* line instead carries a wild-type sequence: KM.) The *App^G–F^* model would be appropriate for use in preclinical studies of β-secretase inhibitors without the interference of the Swedish mutation.

In addition to *App^NL–F^* and *App^NL–G–F^* models, *App* knock-in mice devoid of the Swedish mutations (*App^G–F^* mice) have been recently developed, in which the Swedish mutations (NL) were replaced by a wild-type sequence (KM) (Figure 3 and Table 2). The *App^G–F^* mice are more suitable for preclinical studies of β-secretase inhibition given that the Swedish mutation affects the reactivity of APP to β-site amyloid precursor protein cleaving enzyme 1 (BACE1) and most AD patients do not carry Swedish mutations (Watamura et al., 2021b).

**TABLE 2:** List of mutant mice that are and will be made available to the research community.

Strains	Gene(s)	Modification Information	Availability*^5^	References*^5^
*App^NL^* KI	*App* *^1^	KM670/671NL (Swedish)	RBRC*^6^ (RBRC06342)	Saito et al., 2014
*App^NL–F^* KI	*App* *^1^	KM670/671NL (Swedish), I716F (Iberian/Beyreuther)	RBRC (RBRC06343)	Saito et al., 2014
*App^NL–G–F^* KI	*App* *^1^	KM670/671NL (Swedish), E693G (Arctic), I716F (Iberian/Beyreuther)	RBRC (RBRC06344)	Saito et al., 2014
*App^G–F^* KI	*App* *^1^	E693G (Arctic), I716F (Iberian/Beyreuther)	Soon to be available from RBRC	Watamura et al., 2021b
*App^huAβ^* KI	*App* *^1^	No mutation (humanized Aβ sequence)	Soon to be available from RBRC	Watamura et al., 2021b
*Psen1^P436S^* KI	Psen1*^2^	P436S	Available*^7^	Sasaguri et al., 2018
*Psen1^P117L/A^* KI	*Psen1* *^3^	P117L/A	Available*^7^	Sasaguri et al., 2018; Sato et al., 2021
*App^NL–F^* KI/ *Psen1^P117L^* KI	App*^1^, Psen1*^3^	*App*: KM670/671NL, I716F Psen1: P117L	Soon to be available from RBRC	Sato et al., 2021
*MAPT* KI	*MAPT* ^*4^	Humanization of the *Mapt* gene	RBRC (RBRC09995)	Hashimoto et al., 2019; Saito et al., 2019
*App^NL^* KI/*hMAPT* KI	App*^1^, MAPT*^4^	*App*: KM670/671NL *MAPT*: No mutation	RBRC (RBRC10041)	Saito et al., 2019
*App^NL–F^* KI/*hMAPT* KI	App*^1^, MAPT*^4^	*App*: KM670/671NL, I716F, *MAPT*: No mutation	RBRC (RBRC10042)	Saito et al., 2019
*App^NL–G–F^* KI*/hMAPT* KI	App*^1^, MAPT*^4^	*App*: KM670/671NL E693G, I716F, *MAPT*: No mutation	RBRC (RBRC10043)	Saito et al., 2019
*hMAPT^P301L^* KI	*MAPT* *^4^	P301L	Available*^7^	Watamura et al., 2021b
*hMAPT^P301S^* KI	*MAPT* *^4^	P301S	Available*^7^	Watamura et al., 2021b
*hMAPT^P301V^* KI	*MAPT* *^4^	P301V	Available*^7^	Watamura et al., 2021b
*hMAPT^Intron10+3 G>A^* KI	*MAPT* *^4^	Intron10 + 3 G > A	Available*^7^	Watamura et al., 2021b
*hMAPT^P301L; Intron10+3 G>A^* KI	*MAPT* *^4^	P301L; Intron10 + 3 G > A	Available*^7^	Watamura et al., 2021b
*hMAPT^P301S; Intron10+3 G>A^* KI	*MAPT* *^4^	P301S; Intron10 + 3 G > A	Available*^7^	Watamura et al., 2021b
*hMAPT^S305N; Intron10+3 G>A^* KI	*MAPT* *^4^	S305N; Intron10 + 3 G > A	Available*^7^	Watamura et al., 2021b

*^1^Knock-in of APP sequence (from intron 15 to intron 17) including a humanized Aβ region.

*^2^The mutation was introduced into the murine Psen1 gene by using Base Editor (BE) or Target-AID.

*^3^The mutation was introduced into the murine Psen1 gene by using VQR-BE.

*^4^Replaced the entire genomic sequence of the murine Mapt gene (from exon 1 to exon 14) with the human MAPT gene from the ATG codon of exon 1 to the 3′-UTR.

*^5^As of September 30, 2021.

*^6^RIKEN BioResource Research Center (https://web.brc.riken.jp/en/).

*^7^All strains are available through TCS (takaomi.saido@riken.jp).

Despite the advantages mentioned above, the *App^NL–G–F^* line is not suitable for investigating the metabolism, clearance or deposition of Aβ because the Arctic mutation present in the middle of the Aβ sequence results in an Aβ that is resistant to proteolytic degradation (Tsubuki et al., 2003) and susceptible to aggregation (Nilsberth et al., 2001). Moreover, this model is not suitable for use in preclinical immunotherapy studies due to its affinity for anti-Aβ antibodies, even in the presence of guanidine hydrochloride (GuHCl) (Saito et al., 2014). The Arctic mutation may also directly or indirectly interfere with interactions between Aβ deposition and the apolipoprotein E genotype (Morishima-Kawashima et al., 2000), although there is no experimental evidence for this. In contrast, the *App^NL–F^* line accumulates wild-type human Aβ, but it may take up to 18 months for the pathology to become sufficiently evident for investigational studies to be carried out (Saito et al., 2014), which is too long for researchers to wait in a practical sense. Therefore, a new mouse model that accumulates wild-type human Aβ as quickly as the *App^NL–G–F^* model, but did not depend on the presence of the Arctic mutation was desired.

**FIGURE 4:**
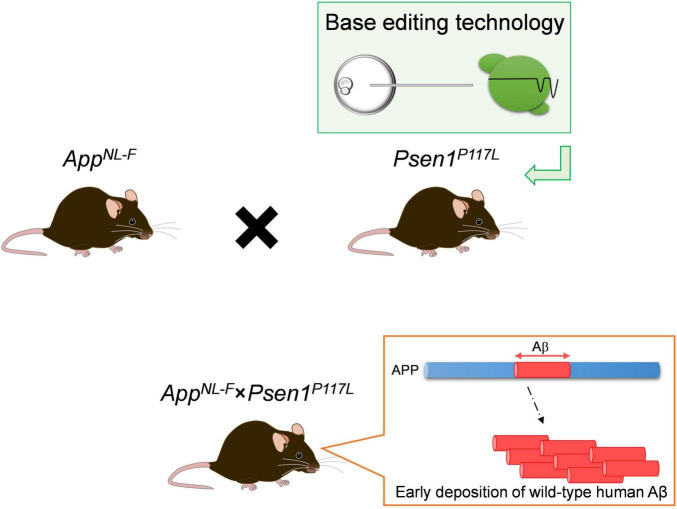
Scheme of *App*^NL–F^** × *Psen1*^*P*117*L*^ double-mutant mice. For the generation of the double-mutant mice, the *App^NL–F^* line was crossbred with the *Psen1*^*P*117*L*^ line whose mutation was introduced in the endogenous *Psen1* gene utilizing base editing technology. The synergistic effects of the pathogenic mutations in the *App* and *Psen1* genes strongly accelerates the deposition of wild-type human Aβ in mouse brains.

**FIGURE 5:**
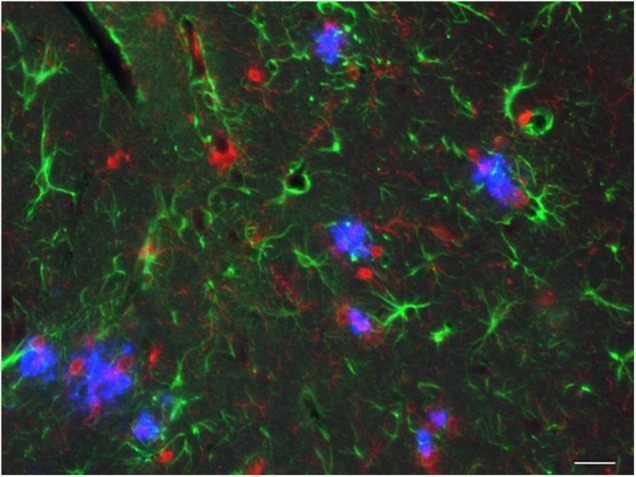
AD pathology in the hippocampus of a 3rd generation model mouse. A 12-month-old *App^NL–F^* X *Psen1*^*P*117*L/WT*^ mouse was analyzed by immunohistochemistry. Blue: Aβ plaques; red: microglia; green: astrocytes. The bar indicates 25 μm.

To achieve this, the heterozygous *Psen1*^*P*117*L/WT*^ mutant line (*Psen1*^*P*117*L*^) which, of the several *Psen1* mutants, exhibits the largest increase in Aβ_42_/Aβ_40_ ratio in the brain (Sasaguri et al., 2018) was utilized. The *Psen1*^*P*117*L*^ line was generated by base editing technology (Komor et al., 2016). The *App^NL–F^* mice were crossed with *Psen1*^*P*117*L*^ mice, despite it being unclear whether their pathogenic effects, both of which act on the γ-cleavage of CTF-β, would be additive or not *in vivo* (Figure 4). The pathological phenotypes of *App^NL–F^* mice were markedly enhanced in a synergistic manner with the *Psen1*^*P*117*L*^ mutation (Sato et al., 2021), with *App^NL–F^* X *Psen1*^*P*117*L/WT*^ mice showing a more aggressive cored plaque pathology and neuroinflammation than the *App^NL–G–F^* mice (Figure 5). These double mutant mice (**3rd generation model**) will likely become highly relevant tools for examining the pathologic mechanisms upstream of Aβ deposition. Moreover, these mice can be highly useful for the preclinical screening of disease-modifying therapy candidates promoting Aβ degradation or disaggregation, without the added concern associated with artificial effects caused by the Arctic mutation. We expect the double homozygous line, *App^NL–F^* X *Psen1*^*P*117*L/P*117*L*^, to exhibit an even more-aggressive pathology. In any case, the *App^NL–F^* X *Psen1*^*P*117*L*^ mice are superior to the *App^NL–G–F^* mice or the *App*^NL–G–I^** mice (Xia et al., 2021) for universal and unbiased drug screening particularly because the Aβ-degrading enzyme, neprilysin (NEP: Neutral endopeptidase), has become a therapeutic target. The *App^NL–G–I^* mice are similarly designed as the *App^NL–G–F^* mice, in which the Beyreuther/Iberian mutation was replaced by the Austrian mutation (Kumar-Singh et al., 2000). The characteristics of the *App* knock-in mouse lines are listed in Table 3.

**TABLE 3:** Characteristics of the *App* knock-in mouse lines.

	Stain	Gene mutations		Genetic background	Aβ plaques (first appearance)	Tangles	Neuronal loss	Cognitive impairment	Strengths	Weaknesses
		*App*	*Psen1*							
Single *App* knock-in	*App* ^*huA*β^	Humanized Aβ	–	C57BL/6J	–	NR	NR	NR	A control for other models	–
	*App^NL^*	Humanized Aβ KM670/671NL	–	C57BL/6J	–	–	–	–	A control for other models	No amyloid pathology No cognitive deficits Increased CTF-β
	*App^NL–F^*	Humanized Aβ KM670/671NL I716F	–	C57BL/6J	6 months	–	–	18 months	Deposition of wild type human Aβ	Long time required for amyloid pathology and cognitive deficits Increased CTF-β
	*App^NL–G–F^*	Humanized Aβ KM670/671NL E693G I716F	–	C57BL/6J	2 months	–	–	6 months	Early appearance of amyloid pathology	The Arctic mutation inside the Aβ sequence Increased CTF-β
	*App^G–F^*	Humanized Aβ E693G I716F	–	C57BL/6J	4 months	NR	NR	NR	Absence of the Swedish mutation No increase of CTF-β	The Arctic mutation inside the Aβ sequence
*App* and *Psen1* double mutant	*App*^NL–F^* Psen1*^P117L^**	Humanized Aβ KM670/671NL I716F	P117L	C57BL/6J	3 months	NR	NR	NR	Early appearance of amyloid pathology Deposition of wild type human Aβ	Mutations in both *App* and *Psen1* genes

*The Swedish mutations; KM670/671NL.*

*The Iberian/Beyreuther mutation; I716F.*

*The Arctic mutation; E693G.*

*NR denotes data not reported.*

## Precautions Regarding the Utility of *App* Knock-In Mice

There are several precautions to be aware of to make the best use of the *App* knock-in mice.

### Nomenclature

A number of the *App* knock-in mouse users use incorrect nomenclature such as APP-NLF, APP*^NLF^* and *APP^NL–F^* instead of the *App^NL–F^* mice, which accords with international rules of standard genomic nomenclature. Genetic names always need to be italicized.

### Line-Ups and Biochemical Analyses

**FIGURE 6:**
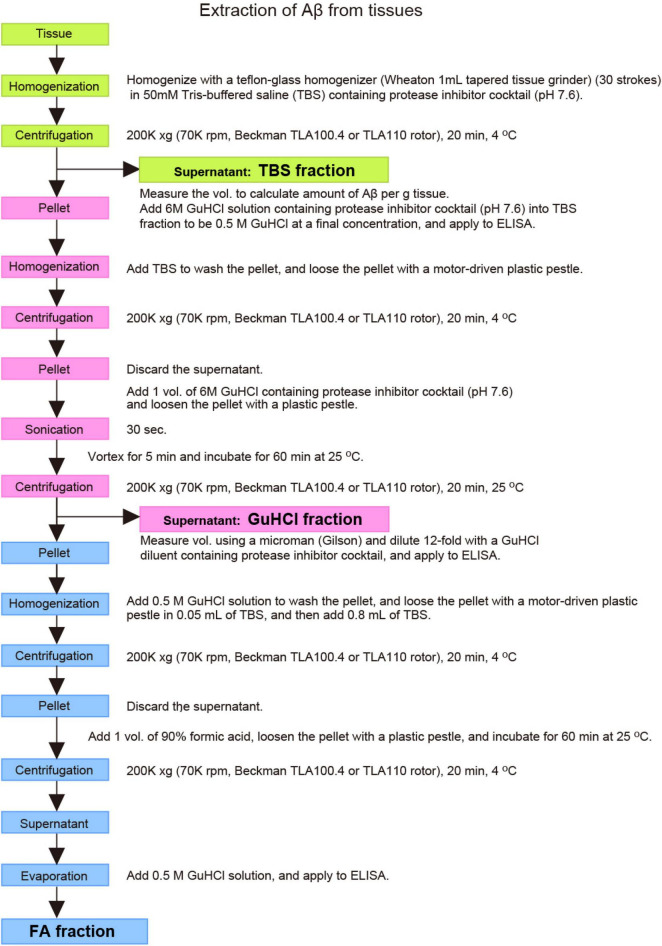
Outlined protocols for extraction and quantification of Aβ from tissues. See text for detailed explanation.

Approximately 20 lines of mutant mice, published or unpublished, can currently or in the very near future be provided to academic and not-for-profit researchers for non-commercial research in a timely fashion with minimum restrictions (Table 2; Saito et al., 2014; Sasaguri et al., 2018). It is also recommended that scientists use optimized protocols for isolating Aβ from animal brain as previously described (Iwata et al., 2005; Figure 6). The method allows the most sensitive quantification of both soluble and insoluble Aβ with the smallest protocol deviations.

### Maintaining Mouse Lines on a Clean C57BL6/J Background

In most cases, the knock-in mice are used in a homozygous state to accelerate the generation of pathological and behavioral phenotypes. However, the number of recessive mutations increases over time if the breeding of mice is maintained in this way. It is necessary therefore to back-cross heterozygous mutant mice with the wild-type C57B6/J mice to remove these extraneous mutations, preferentially for 5–10 generations at an interval of 5–10 generations. Those groups dealing with poor reproductive output of mice due to their extremely inbred nature can contact RIKEN BioResource Research Center (email: animal.brc@riken.jp), a national mouse repository of Japan (Mizuno-Iijima et al., 2021), to renew their strains.

### Choosing Appropriate Anti-Aβ Antibodies

**FIGURE 7:**
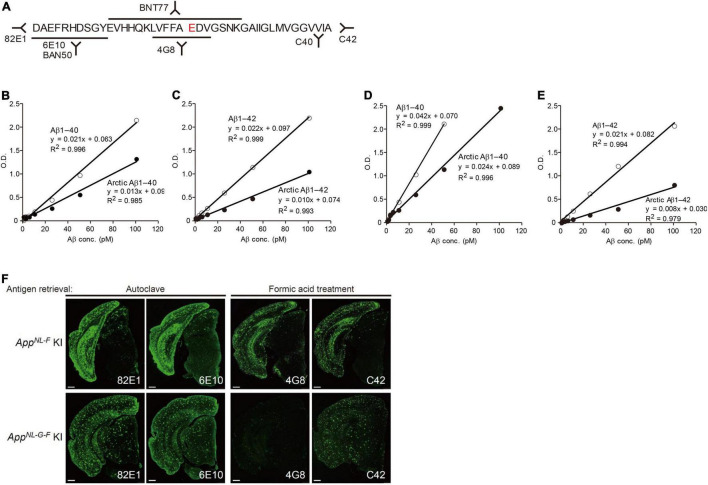
Reactivity of different antibodies to Arctic Aβ in *App^NL–G–F^* mice. **(A)** Epitope map of anti-Aβ antibodies. **(B,C)** Quantification of Arctic Aβ species using BNT77 as a capture antibody. BNT77 binds to the mid-portion of Aβ [see epitope map **(A)**]. A sandwich ELISA kit (Wako, Japan) was used to quantify Aβx-40 **(C)** and Aβx-42 **(D)**, respectively. **(D,E)** Quantification of Arctic Aβ species using BAN50 as a capture antibody. BAN50 binds to the N-terminal region of Aβ [see epitope map **(A)**]. A sandwich ELISA kit (Wako, Japan) was used to quantify Aβx-40 **(D)** and Aβx-42 **(E)**, respectively. BNT77 and BAN50 captured Arctic Aβ more weakly than wild-type Aβ. **(F)** Immunohistochemistry using various anti-Aβ antibodies. Brain sections derived from *App^NL–F^* mice (24 months old) were immunostained using antibodies with different epitopes after antigen retrieval as indicated (upper panels); those of *App^NL–G–F^* mice (9 months old) were similarly immunostained (lower panels). Scale bars represent 500 μm.

It was previously indicated that some anti-Aβ antibodies are inappropriate for biochemically and pathologically detecting the Arctic Aβ produced by *App^NL–G–F^* mice (Saito et al., 2014; Figure 7). Note that the *App^NL–G–F^* is the most frequently used model because it recapitulates Aβ pathology and neuroinflammation much faster than other lines. This applies to all other transgenic and knock-in mice that carry intra-Aβ mutations, including the Arctic and Dutch mutations (Van Broeckhoven et al., 1990; Li et al., 2014). In contrast, there is no restriction regarding use of antibodies for the *App^NL–F^* X *Psen1*^*P*117*L*^ mice.

### Preclinical Nature of Behaviors

In our experience, the most sensitive and reproducible test involves contextual fear conditioning, although its irreversible nature can be problematic. In a more complex sense, the application of a multi-task paradigm such as ***IntelliCage*** (NewBehavior AG, Zurich, Switzerland) would be more informative (Codita et al., 2010; Masuda et al., 2016). We recently showed that the mouse genotypes can be predicted from their behavioral parameters by machine learning (Sutoko et al., 2021). It should be emphasized that the *App* knock-in mice are models of preclinical AD because the *App* knock-in mice, like all the APP and APP/PS1 transgenic mice, do not recapitulate tau pathology alone (Sasaguri et al., 2017). Consistently, we observe only mild cognitive decline in these mice. In contrast, the *App* knock-in mice crossbred with mutant *MAPT* knock-in mice exhibited accelerated tau pathology (Table 2).

## A New Model of Cerebral Amyloid Angiopathy

Most AD patients exhibit parenchymal and vascular Aβ deposition in the brains, and both pathologies seem to be driven by impaired Aβ clearance within the interstitial fluid and perivascular drainage pathways (Greenberg et al., 2020). Iliff et al. (2012) injected fluorescent tracers into Tie2-GFP:NG2-DsRed double reporter mice, which express GFP in all cerebral blood vessels and DsRed in perivascular cells, and successfully observed glymphatic pathway; subarachnoid CSF influx into the brain parenchyma and bulk ISF solute clearance from the parenchyma within the perivascular spaces. Importantly, in AD model mice, glymphatic CSF influx is reduced and the clearance of Aβ is severely impaired (Peng et al., 2016). Impaired glymphatic pathway may contribute to the deposition of Aβ in the blood vessels of the brain, cerebral amyloid angiopathy (CAA). Although CAA is profoundly observed in most AD patients (Brenowitz et al., 2015), limited model mice, such as those with Dutch/Iowa mutation, exhibit apparent CAA, thus making it difficult to determine how CAA contributes to the pathogenesis of sporadic AD. Notably, human vascular endothelial cells express significant level of APP770 and human plasma contains ∼100 ng/ml of sAPP770 (Kitazume et al., 2010). Since peripheral blood cells other than platelet do not express APP, and platelets release sAPP770 upon their activation (Miura et al., 2020), it is considered that plasma sAPP770 is mostly derived from endothelial APP770. Because in rodents plasma sAPP is a markedly lower (∼100 pg/ml) than that of humans (Kitazume et al., 2012), it’s possible that low level of endothelial APP expression in mice could be one of the reasons that *App* knock-in mice exhibit mild CAA pathology. To overcome this, a mouse line that specifically expresses human APP770 in endothelial cells has just been generated (unpublished). In brief, floxed hAPP770NL mice under the CMV early enhancer/chicken β-actin promoter were first generated. These mice were then crossed with Tie2-Cre mice, in which the Tie2 promoter directs the expression of Cre recombinase in the endothelial cells to obtain double transgenic (Tg) mice.

*App* knock-in mouse models were previously produced by Li et al. (2014) who used multiple pathogenic mutations. These mice carry the Swedish (K670N/M671L), Dutch (E693Q), and London (V717I) mutations with the humanized Aβ sequence. The Dutch mutation results in an intensive CAA pathology in humans, thereby causing brain hemorrhage and early mortality (Levy et al., 1990; Van Broeckhoven et al., 1990). This mutation is therefore not specifically responsible for causing FAD. These mice did not develop prominent Aβ deposits over their lifespan, but when they were crossbred with *Psen1*^*M*146*V*^ knock-in mice, an age-dependent deposition of Aβ was seen in the resultant double knock-in mice. The deposition of Aβ was detected not only in the parenchyma of the cerebral cortex but also in the cerebral vasculature, similar to that seen in CAA in humans. Double knock-in mice that did not have the Dutch mutation exhibited virtually no vascular pathology. In this way, if the authors had used the Beyreuther/Iberian or Austrian mutation instead of the London mutation in the mouse *App* gene then they probably would not have had to introduce the *Psen1* knock-in mice. Knock-in mice harboring the Dutch mutation can still serve as relevant models for CAA; however they may not be appropriate for examining the effect of immunotherapy on CAA because the Dutch mutation is present in the middle of the Aβ sequence.

## Neprilysin-Sensitive Amyloidogenic Aβ as a Probable Cause for Sporadic Alzheimer’s Disease

NEP and insulin-degrading enzyme (IDE) are considered the two major catabolic enzymes that degrade Aβ (Qiu et al., 1998; Iwata et al., 2000, 2001; Farris et al., 2003; Leissring et al., 2003). NEP is capable of degrading both soluble and insoluble Aβ (Iwata et al., 2000, 2001; Huang et al., 2006), but it is not clearly shown whether endogenous IDE could degrade insoluble Aβ in the mouse brains (Farris et al., 2003), rather IDE appears to be involved in metabolism of AICD. Unfortunately, their roles in Aβ metabolism *in vivo* have never been compared in an impartial and side-by-side manner. Once double mutants crossbred single *App* knock-in mice with NEP (*Mme*) KO mice and with IDE (*Ide*) KO mice were analyzed in detail for their biochemical properties and Aβ pathology properties, it would be clear their distinct roles in APP metabolism and the AD pathogenesis.

Further to the above, a deficiency of NEP had no significant impact on the levels of various neuropeptides (Sasaguri et al., 2018) as well as enkephalins (Saria et al., 1997) that are well known to be *in vitro* substrates for NEP (Turner et al., 1996, 2000, 2001; Turner and Nalivaeva, 2006) in the cerebral cortex and hippocampal formation of mice, presumably because NEP is mainly expressed in secretory vesicles and on the presynaptic membranes of excitatory neurons (Iwata et al., 2002, 2004, 2013), while most if not all neuropeptides are secreted from inhibitory neurons. This argues against the concern that NEP up-regulation for the treatment of preclinical AD would reduce the levels of these neuropeptides. These findings also indicate that NEP relatively selectively degrades Aβ in the brain. Whereas familial AD (FAD) is unambiguously caused by an increased anabolism of Aβ, and of Aβ_42_ and Aβ_43_ in particular (Selkoe and Hardy, 2016), the anabolism of Aβ appears unaffected prior to its deposition in the brain that subsequently leads to the onset of sporadic AD (SAD). These observations suggest that NEP-sensitive amyloidogenic Aβ likely plays a primary pathogenic role in the etiology of SAD. Our findings are consistent with the aging-dependent decline of NEP expression in the human brain and with recent genome-wide association studies (GWAS) indicating that variants of the gene encoding NEP (*MME*) are associated with the risk of SAD development (Bellenguez et al., 2020). Taken together, our results imply that the aging-associated decrease in NEP expression is a primary cause of SAD and could thus be a target for the treatment of preclinical AD once other factors such as apolipoprotein E genotypes have also been considered.

## Regulation of Aβ Metabolism *via* Somatostatin Receptor Subtypes Through Modulation of Nep Activity

Since NEP is a major Aβ-degrading enzyme and it is downregulated upon aging, its decreased levels in the brain will most likely lead to increased Aβ levels (Yasojima et al., 2001; Carpentier et al., 2002; Iwata et al., 2002; Maruyama et al., 2005; Hellström-Lindahl et al., 2008). NEP is regulated by the neuropeptide somatostatin (Saito et al., 2005). Somatostatin, which binds to somatostatin receptors, is also decreased upon aging and in AD possibly due to loss of somatostatin-positive interneurons (Davies et al., 1980; Beal et al., 1985; Bergström et al., 1991; Hayashi et al., 1997; van de Nes et al., 2002; Lu et al., 2004; Gahete et al., 2010). Somatostatin, which was first identified to regulate secretion of growth hormone from pituitary, has been traditionally abbreviated as SRIF (somatotropin release-inhibiting factor) (Møller et al., 2003), so we will keep to this nomenclature in this review. SST_1_, SST_2_, SST_3_, SST_4_, and SST_5_ are used to express somatostatin receptor subtypes 1–5. Interestingly, mutations in SRIF are linked to AD (Vepsäläinen et al., 2007). By using a combination of *in vitro* and *in vivo* approaches to identify the subtype specificity of the five SSTs expressed in the brain and considered to be involved in the regulation of NEP. We would like to emphasize that it is necessary to use a co-culture system of primary neurons from the cortex, hippocampus, and striatum for *in vitro* experiments (Kakiya et al., 2012; Nilsson et al., 2020; Watamura et al., 2021a).

**FIGURE 8:**
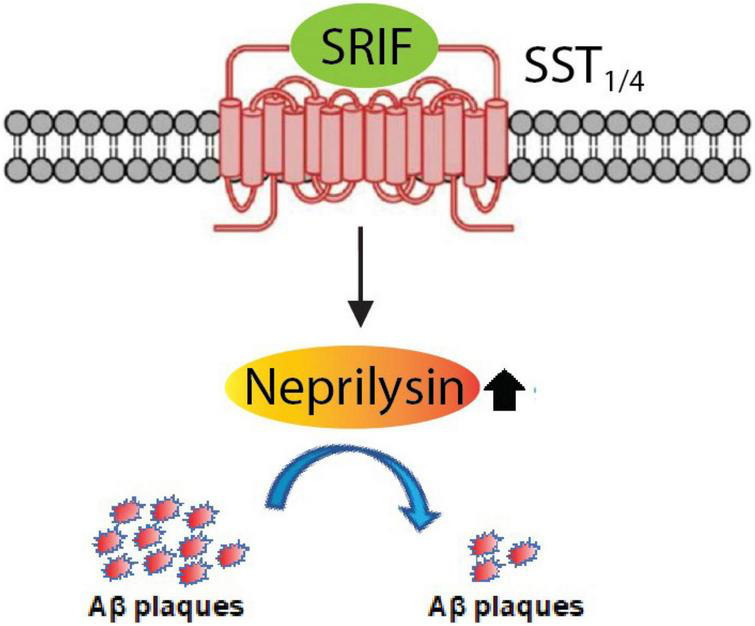
Somatostatin receptor subtypes 1 and 4 (SST_1/4_) regulate the Aβ-degrading enzyme NEP. The neuropeptide somatostatin (SRIF) was identified as a regulator of NEP activity through *in vitro* screening. Subsequent analysis of the effect of genetic deletion of somatostatin receptor (SST) subtypes in mice revealed that SST_1_ and SST_4_ regulate NEP in a redundant manner. This was further confirmed by concurrently deleting SST_1_ and SST_4_ in *App* KI mice, which aggravated the Aβ pathology. SST_1/4_ can be either a combination of SST_1_ and SST_4_ homodimers or an SST_1_/SST_4_ heterodimer.

Using a battery of *Sst* double knockout (dKO) mice, we found that NEP is regulated by SST_1_ and SST_4_ in a redundant manner. *Sst*_1_ and *Sst*_4_ dKO mice exhibit a specific decrease of presynaptic NEP in the Lacunosum molecular layer. Moreover, a genetic deficiency of *Sst*_1_ and *Sst*_4_ in the *App* knock-in mice aggravated the Aβ pathology in the hippocampus. As a first proof of concept towards an Aβ-lowering strategy involving NEP, a treatment with an agonist selective for SST_1_ and SST_4_ could ameliorates the Aβ pathology and improves cognitive outcomes in the *App* knock-in AD mouse model as schematized in Figure 8 (Nilsson et al., 2020). These results indicate that a combination of SST_1_ and SST_4_ homodimers or the SST_1_ and SST_4_ heterodimer may become a target for pharmaceutical intervention to treat preclinical AD. Of note, the expression of SRIF in human brain declines with aging and in AD (Davies et al., 1980; Lu et al., 2004) and may causally contribute to AD pathogenesis *via* reduction of NEP activity/expression.

## SRIF-Evoked Aβ Catabolism in the Brain: Mechanistic Involvement of the α-Endosulfine-K_*ATP*_ Channel Pathway

Although SRIF is known to regulate Aβ catabolism by enhancing NEP-catalyzed proteolytic degradation, the mechanism by which SRIF actually regulates NEP activity is yet to be fully elucidated. Proteomic analyses enabled α-endosulfine (ENSA), an endogenous ligand of the ATP-sensitive potassium (K_*ATP*_) channel, to be identified as a negative regulator of NEP downstream of SRIF signaling (Watamura et al., 2021a). The expression of ENSA is significantly increased in AD mouse models and in patients with AD. In addition, NEP directly contributes to the degradation of ENSA, suggesting a substrate-dependent feedback loop regulating NEP activity.

**FIGURE 9:**
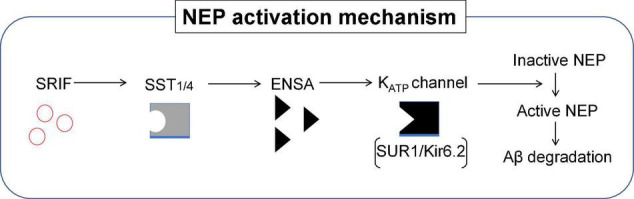
Role of ENSA in the regulation of NEP activity. Schematic illustration of the mechanism describing NEP activity in the brain. ENSA, a downstream protein of SST-SST_1/4_ signaling, plays a role as a ligand of the K_*ATP*_ channel composed of sulfonylurea receptor subunit 1 (SUR1) and inwardly rectifying K^+^ channel 6.2 (Kir6.2), resulting in the activation of NEP. SST_1/4_ can be either a combination of SST_1_ and SST_4_ homodimers or an SST_1_/SST_4_ heterodimer.

It was also discovered the specific K_*ATP*_ channel subtype [sulfonylurea receptor subunit 1 (SUR1) and inwardly rectifying K^+^ channel 6.2 (Kir6.2)] that modulates NEP activity, resulting in altered Aβ levels in the brain. Pharmacological intervention targeting this particular K_*ATP*_ channel by diazoxide attenuated Aβ deposition, with impaired memory function rescued *via* the NEP activation in our AD mouse model. These findings provide a mechanism explaining the molecular link between K_*ATP*_ channels and NEP activation. They also provide new insights into how ENSA and the K_*ATP*_ channel could profile as a new therapeutic target for lowering Aβ and thus provide an alternative strategy to prevent AD. Figure 9 summarizes the NEP activation mechanism that involves SRIF receptor subtypes, ENSA and K_*ATP*_ channel involvement.

## Humanization of the Entire Murine *Mapt* Gene to Generate *hMAPT* Knock-In Mice

To date, most if not all, mouse models of tauopathy have been unable to recapitulate the tau pathology without overexpressing mutant human tau protein. As a novel *in vivo* platform for studying human tauopathy, human *MAPT* knock-in mice have been developed in which the entire *Mapt* gene including all exons and introns are humanized (Hashimoto et al., 2019). In each strain, the *MAPT* and *Mapt* genes encoded human and murine tau proteins, respectively. This was done by crossing *MAPT* knock-in mice with single *App* knock-in mice in order to study the role of the Aβ-tau axis in the etiology of AD. The double knock-in mice exhibited a more pronounced tau phosphorylation status than single *MAPT* knock-in mice but lacked evidence of tau pathology and neurodegeneration (in a manner similar to that of single *App* knock-in mice) even after waiting until the mice were 24 months old.

**FIGURE 10:**
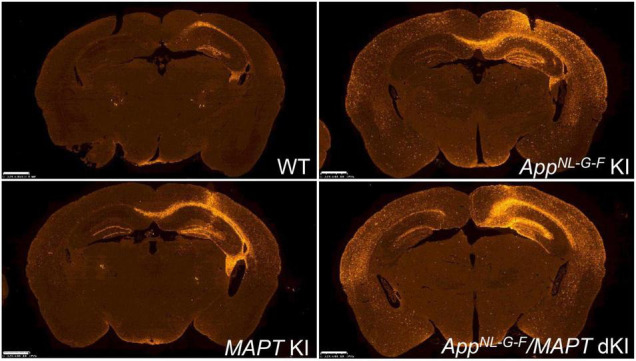
Propagation of AD-tau in mouse brains. Propagation of tau in each mouse line was observed 3 months after AD-tau injection. Brain sections were immunostained with AT8 (red). Humanization of the host animal tau affects the transmission of the pathogenic agents. *App*^NL–G–F^*/MAPT* dKI mice exhibited greater pathological propagation than *App^NL–G–F^* KI mice.

In both the absence and presence of Aβ amyloidosis, the tau humanization has been found to significantly accelerate the propagation of AD brain-derived pathological tau (Figure 10; Saito et al., 2019). Tau accumulation was intensified in the latter case and closely associated with dystrophic neurites, consistently showing that Aβ amyloidosis affects tau pathology. These results indicated that pathological human tau interacted better with human tau than with murine tau, and suggest the presence of a species-defined preference between the pathogenic proteins. The *MAPT* knock-in mice also facilitate the investigation of behavioral properties and of human tau characteristics in living animal models. In addition, mutant *MAPT* knock-in mice carrying various pathogenic mutations have been generated (Table 2). These mice exhibit aging-dependent tau aggregation and cognitive impairment in a manner accelerated by Aβ pathology and are being provided to the research community upon request.

## The Role of Capon in Tau Pathology-Mediated Toxicity

Pathological tau causes synaptic dysfunction and loss of synapses. One of promising molecules that mediates tau pathology-induced neurotoxicity is *N*-methyl-D-aspartate receptor (NMDAR). For example, tau accumulation disturbs synaptic plasticity through JAK2/STAT1-mediated suppression of NMDAR (Li et al., 2019). Phosphorylation of tau at Tyr18, which is mediated by the tyrosine kinase Fyn, enhances NMDAR-dependent excitotoxicity (Guo et al., 2020). Several reports indicated that glutamate-induced excitotoxicity was prevented by downmodulation of tau (Roberson et al., 2007) (Ittner et al., 2010). We also previously identified a NMDAR-related molecule as a tau binding protein which is involved in tau pathology- induced neurodegeneration.

To elucidate key molecules underlying tau accumulation-induced neurodegeneration, a comprehensive screening of tau-interacting proteins (tau interactome) was constructed. Tau-binding proteins were isolated by immunoprecipitation-LC-MS/MS (IP-MS) using a Flag-tag antibody and wild-type tau Tg (wtau-Tg) mice, which is expressing human tau tagged with a flag epitope (Kimura et al., 2007). Considering that tau is a microtubule-binding protein, we validated the methods used to generate the tau interactome by identifying the tubulin beta-4A chain as one of the tau-binding proteins.

Of the many proteins identified in the tau interactome, we focused on carboxy-terminal PDZ ligand of neuronal nitric oxide synthase (CAPON) (Hashimoto et al., 2019), which is an adaptor protein of neuronal nitric oxide synthase (nNOS). CAPON acts as an enzyme for the production of nitric oxide (NO) and is involved in NMDAR-mediated excitotoxicity (Jaffrey et al., 1998). It is thought to recruit substrates to nNOS and facilitate their NO-mediated modification through protein-protein interactions (Jaffrey et al., 1998). The presence of CAPON polymorphisms associated with schizophrenia and other psychiatric disorders has been reported in several studies (Brzustowicz, 2008; Freudenberg et al., 2015). Moreover, CAPON was shown to positively regulate spine density (Richier et al., 2010) and to regulate neuronal cell death downstream of the NMDAR (Li et al., 2013). These findings suggest that CAPON contributes to neurotransmission and neuronal excitotoxicity. In addition, one report showed that CAPON is upregulated in CA1 pyramidal cells in the AD brain (Hashimoto et al., 2012), implying that CAPON may play an important role in the pathogenesis of AD. The mechanism(s) underlying these effects nevertheless remain(s) unknown.

**FIGURE 11:**
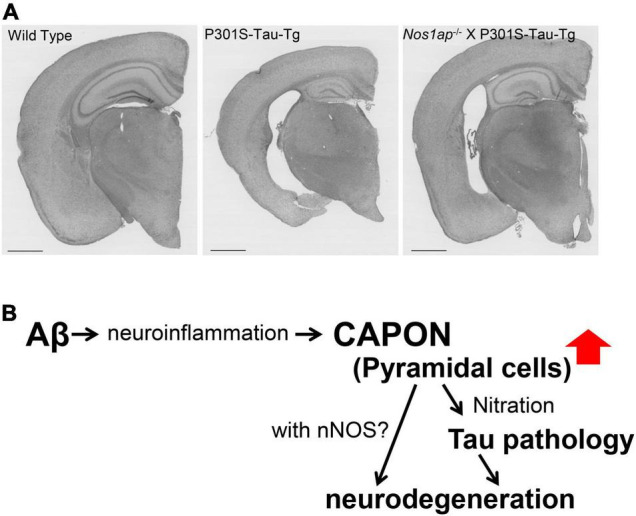
Functions of CAPON in neurodegeneration. **(A)** Brain sections from WT, P301S-Tau-Tg or *nos1ap*-/-/P301S-Tau-Tg mice stained by the conventional method using hematoxylin and eosin (H&E). A CAPON (*Nos1ap*) deficiency restores AD-related pathological phenotypes in P301S-Tau-Tg mice. **(B)** Scheme of CAPON action. Aβ pathology elevates the level and localization of CAPON in hippocampal pyramidal cells. CAPON-induced neuronal cell death is closely associated with the pathological tau protein, although there appears to be a tau-independent mechanism in play as well.

To further elucidate the effects of CAPON on AD pathology, we introduced CAPON cDNA into the brains of *App^NL–G–F^* and *App^NL–G–F^* X *MAPT* (hTau) double-KI mice using a newly developed adeno-associated virus (AAV)-mediated approach. We analyzed the effects of human tau protein as it is known that the hTau-KI mouse expresses an endogenous level of WT human tau. These experiments revealed that CAPON expression facilitates hippocampal atrophy in conjunction with neuronal cell death, and that a deficiency of CAPON in the P301S-Tau-Tg tauopathy mouse model suppressed tau pathology and neurodegeneration (Figure 11). From our results, an intervention in the interaction between CAPON-tau or CAPON-nNOS could be a new approach for the treatment of AD.

## Generation of Non-Human Primate Models of Familial Alzheimer’s Disease

**FIGURE 12:**
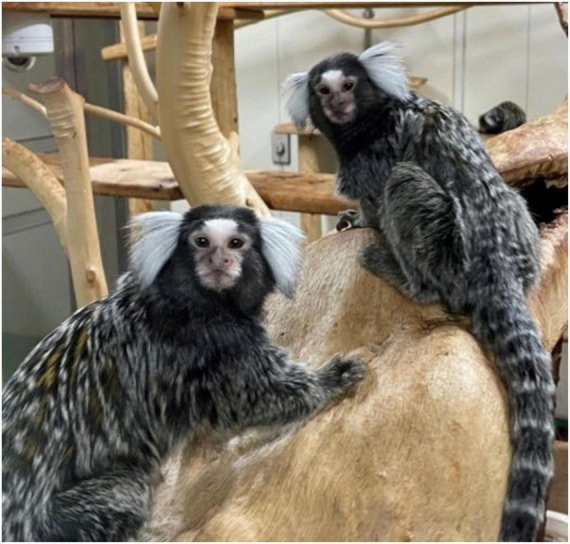
Photograph of common marmosets (*Callithrix jacchus*). The photo shows members of captive common marmoset family. Their small body size, fecundity, and high cognitive functions are a suitable model for neuroscience. The photograph of marmosets was taken by WK at CIEA.

Common marmosets (marmosets, *Callithrix jacchus*) are small non-human primates that belong to the New World Primates (Figure 12; Mansfield, 2003). They have been increasingly utilized in neuroscience because of advantages that were observed over other research primates (Okano, 2021; Park and Sasaki, 2021). Marmosets possess physiological functions, brain structures and complex cognitive/social behaviors similar to those of humans; they communicate mainly *via* visual and auditory measures. In association with AD research, the amino acid sequence of Aβ in marmosets is identical to that of humans, with aged wild-type marmosets starting to accumulate Aβ from 7 years of age or even earlier (Geula et al., 2002; Rodriguez-Callejas et al., 2016). In addition, adolescent marmosets exhibit tau hyperphosphorylation, but not neurofibrillary tangle formation, in the brain that increases with aging (Rodriguez-Callejas et al., 2016). Their life spans in captivity are as long as 10–15 years, making them suitable for age-related research (Tardif et al., 2011). Their immune systems and metabolic functions resemble those of humans (t Hart and Massacesi, 2009; Tardif et al., 2011) and thus may affect the pathogenic processes related to AD (Ennerfelt and Lukens, 2020; Kellar and Craft, 2020; Rosario et al., 2020). Because sleep disorder is an early clinical symptom of AD (Pyun et al., 2019), it is noteworthy that marmosets share with humans the sleep phases composed of rapid eye movement (REM) and non-REM cycles (Crofts et al., 2001). Among various non-human primate species, the marmoset seems most applicable to genetic manipulation, i.e., generation of designed mutants, for which their high reproductive efficacy is advantageous (Sasaki et al., 2009; Sato et al., 2016; Park and Sasaki, 2021). Furthermore, fecundity characteristics of marmosets, such as a short period of sexual maturity, multiple births, and short gestation interval, are suitable for producing genetically modified disease models (Tardif et al., 2003).

Majority of FAD-causing mutations reside in the *PSEN1* gene (Scearce-Levie et al., 2020). Typically, deletion mutations in exon 9 (Crook et al., 1998; Prihar et al., 1999; Smith et al., 2001; Dumanchin et al., 2006; Le Guennec et al., 2017) or point mutations at the 3′ splice site (acceptor site) of exon 9 in the *PSEN1* gene cause dominantly inherited FAD. The point mutations instigate exon 9 elimination and S290C modification in the corresponding mRNA at the junction sites of exons 8 and 10 *via* the conversion of alternative splicing (Hutton et al., 1996; Kwok et al., 1997; Steiner et al., 1999; Brooks et al., 2003; Blauwendraat et al., 2016). Thus, generation of a marmoset model of AD is set out in which exon 9 of the *PSEN1* gene product is deleted using gene-editing technologies to produce AD marmoset models. Since TALEN exhibited high genome-editing efficacy, generates few off-target effects, and produces little mosaicism, the TALEN would be a suitable tool for producing exon 9 deletion in the *PSEN1* gene (Sato et al., 2016; Zhang et al., 2019). Although it is a non-peer review data, the exon 9 deletion in the *PSEN1* gene that is an AD causing mutation has been successfully introduced into non-human primates by TALEN (Sato et al., 2020).

## Author Contributions

All authors listed have made a substantial, direct, and intellectual contribution to the work, and approved it for publication.

## Conflict of Interest

The authors declare that the research was conducted in the absence of any commercial or financial relationships that could be construed as a potential conflict of interest.

## Publisher’s Note

All claims expressed in this article are solely those of the authors and do not necessarily represent those of their affiliated organizations, or those of the publisher, the editors and the reviewers. Any product that may be evaluated in this article, or claim that may be made by its manufacturer, is not guaranteed or endorsed by the publisher.

## Footnotes

1
http://www.alzforum.org
